# Gestalt approach and cognitive fallacies: mitigating negative recency and positive recency

**DOI:** 10.1007/s10339-025-01309-1

**Published:** 2025-10-14

**Authors:** Yeonho Choi, Kyungil Kim, Lisa K. Son

**Affiliations:** 1 Department of Psychology, The Ohio State University, 221C Lazenby Hall, 1827 Neil Avenue, Columbus, OH 43210 USA; 2 Department of Psychology, Ajou University, Suwon, Republic of Korea; 3 Department of Psychology, Barnard College, New York, USA

**Keywords:** Gestalt approach, Negative recency, positive recency, Law of small numbers

## Abstract

This study examined whether Gestalt-based manipulations can reduce individual’s tendency to follow negative recency (NR) or positive recency (PR) by inducing their perception that events are not related. Two types of manipulation – grouping and closure – were introduced through a series of Coin Prediction Tasks. In the Grouping-based Coin Prediction Task (G-CPT), participants were more likely to exhibit NR or PR tendencies when past outcomes and predictions were presented within the same trial compared to when they were presented in separate trials. In the Closure-based Coin Prediction Task (C-CPT), previous results ending with a streak of a dominant event elicited more NR or PR responses, whereas previous outcomes ending with a non-dominant event reduced these fallacies. Overall, this study provides evidence that Gestalt-based manipulations can mitigate cognitive fallacies. Our findings emphasize the importance of Gestalt framing in probabilistic decision-making contexts. Limitations and directions for future research are also discussed.

## Introduction

In a random sequence, people perceive specific patterns by monitoring previous outcomes (Gaissmaier et al. 2016; Van Prooijen et al. 2018). For example, when betting on a single number on the roulette wheel (i.e., “Straight-up bet”), some people may bet on the “hot” number, that is, the number that was frequently presented in previous trials, while others tend to avoid it (Sundali and Croson 2006). Regardless of the two options, given that a random sequence is independent and thus inherently unpredictable (Burns and Corpus 2004; Wagenaar 1991), such bets would be indicative of a cognitive fallacy. We focused on two cognitive fallacies: *negative recency* (NR; also known as the gambler’s fallacy) involves the mistaken belief that a streak of an outcome will be reversed in the future, and *positive recency* (PR) reflects the irrational expectation that a streak of an outcome will continue.^1^ The current research examined the following: Does the grouping pattern of past results impact the likelihood of succumbing to subsequent fallacies?

Both NR and PR have been explained using the belief in the *law of small numbers*, where people consider a population’s characteristics and believe that such expectations should be applied even to small samples (Kahneman and Tversky 1972; Tversky and Kahneman 1971). For example, individuals might imagine an equal proportion of heads and tails in a fair coin toss to appear in any random sequence (Tversky and Kahneman 1971). Thus, NR can arise when a sequence includes a streak of one event, such as a streak of heads, and, to move towards an equal proportion, expect that the next toss must be tails. On the other hand, sometimes, people perceive a streak of events as evidence that contradicts representativeness (i.e., randomness) of the sample (Ayton and Fischer 2004; Gilovich 1991). Therefore, when a run of one event (e.g., a streak of heads) is observed in a random sequence, individuals may believe the sequence is biased rather than reflecting the characteristics of the population, thereby following PR (Gilovich et al. 1985). In sum, belief in the law of small numbers suggests that two cognitive fallacies can emerge depending on how people perceive the randomness of a sequence: (1) NR can arise when believing the sequence is random (i.e., representative of the population) and (2) PR can occur when believing the sequence is non-random (i.e., not representative of the population).

The law of small numbers has been specified by Rabin’s (2002) mathematical model, which states that people think about sampling in a binary event as if they were drawing balls one by one from an urn containing two types of balls without replacement. For example, if an urn (coin toss) contains five heads and five tails, and one head is drawn, people perceive the probability of tails as higher because four heads and five tails remain. One of the model’s important predictions is that when people do not know the true probability of an outcome, they infer it from the sampled sequence, leading both PR and NR to appear. Specifically, when a short run of an outcome (e.g., HH) occurs, people imagine the urn as being evenly composed of both outcomes. However, as the run length increases (e.g., HHHHH), they instead imagine an urn more heavily composed of a specific outcome (e.g., heads). In other words, NR tends to appear after short runs, whereas PR tends to appear after long runs. This prediction was empirically supported (Asparouhova et al. 2009).

However, Roney and Trick (2003) pointed out that the belief in the law of small numbers fell short of explaining why individuals perceive independent events as being related in the first place. They proposed that people first categorize independent events into a meaningful group based on Gestalt principles, perceiving the events as related rather than separate. This process could then give rise to certain cognitive fallacies, by their beliefs in the law of small numbers (Roney and Trick 2003). In other words, Roney and Trick (2003) argued that cognitive fallacies would not occur if events were, appropriately, perceived as separate. For NR, several studies supported this idea (Gold and Hester 2008; Roney and Sansone 2015; Roney and Trick 2003). For example, Roney and Trick (2003) demonstrated that people exhibited NR when predicting the seventh outcome in the same block after observing the outcome of six coin tosses but not when predicting the first outcome of the next block after observing the same results. This suggests that NR occurs only when “grouping” the past and future into a single meaningful set (e.g., “same block”) rather than perceiving them as separate sets (e.g., “different block”).

Similarly, Roney and Sansone (2015) showed that NR can appear or disappear depending on whether a streak of an event is interrupted by another event. In their study, they observed how participants in the two experimental groups predicted the thirteenth outcome after observing the outcome of 12 tosses. The first eight outcomes (e.g., “HTHHTHHT”) were identical for both groups, but the last four outcomes were differently presented. In the “open run” group, the last outcome was “one non-dominant side followed by a run of the dominant side” (e.g., “THHH”), whereas in the “closed run” group, it was “a run of the dominant side followed by one non-dominant side” (e.g., “HHHT”). Their results showed that only participants in the “open run” group exhibited NR. This suggests that when the run remains unbroken (open), people are likely to perceive it as related to the next outcome. In contrast, when they encounter a “closure” of the run, their expectations for the next one seem to “reset” (Roney and Sansone 2015).

The previous studies clearly showed that NR occurs only when past events and predictions for the future are perceived as related. Furthermore, their findings cannot be explained by the belief in the law of small numbers, as the sequences presented across experimental groups were either identical or had the same proportion (e.g., an 8:4 ratio of events). Given these findings, we aimed to investigate two questions. First, does PR also occur only when past and future events are perceived as connected? Like NR, PR violates the assumption of independence that the probability of an event is constant regardless of previous outcomes. Thus, as suggested by Roney and Trick (2003), we believed that PR would appear when the past seems to be connected with the future event but would disappear when they are perceived as unrelated. Second, do the Gestalt-based manipulations (i.e., grouping and closure of a run) used in the previous studies truly lead to the perception of either a connection between the past and the future or no connection between them? Since the previous studies did not check this directly, we set manipulation checks to assess the effect of their manipulations.

## The current study

This study focused on the following primary research question: Do cognitive fallacies occur only when events are perceived as related? To test this, we proposed two hypotheses:

- H1: The tendency to follow NR will decrease when events are perceived as unrelated (replicating previous studies).
- H2: The tendency to follow PR will decrease when events are perceived as unrelated (a novel question).

### Design & materials

#### Past experience questions

Two experience variables were measured. “Gambling” represented each participant’s gambling experiences in the past. To measure this experience, we used a general question derived from a previous study (Roney and Sansone 2015): “How much gambling experience do you have?” where participants had to answer on a scale from 1 (none) to 5 (a lot). “Probability” represented each participant’s understanding of the law of probability: “How familiar are you with the following terms of the Law of Probability: Expected value, Conditional Probability, and Independence?” Again, participants were asked to answer this question on a scale of 1 (not at all) to 5 (extremely). These measures were used as covariates in the analysis. This was because a higher level of knowledge about the Law of Probability might reduce susceptibility to gambling fallacies (Turner et al. 2022; Williams and Connolly 2006). Furthermore, gambling experience is associated with NR (Roney and Sansone 2015).

#### Grouping-based coin prediction task (G-CPT)

This task was based on the study by Roney and Trick (2003). All participants completed this task across two grouping conditions. In the “same trial” condition, they predicted the outcome of each toss in each trial consisting of seven tosses. However, in the “different trial” condition, they forecasted the outcome in each trial consisting of six tosses. Our primary goal was to observe whether participants followed either NR bias or PR bias when predicting the next outcome after observing the same six toss outcomes (e.g., “HTHTTT”) in both conditions. That is, we examined whether they exhibited cognitive fallacies when predicting the outcome of the seventh toss in the “same trial” condition and the first toss of the next trial in the “different trial” condition. Since the six toss results presented before the prediction were identical in both conditions, we were able to compare differences in their predictions across the conditions. We expected that people would tend to follow cognitive fallacies in the “same trial” condition but not in the “different trial” condition. This was because the meaningful grouping between the previous outcomes and prediction would be easier to form in the “same trial” condition.

While doing so, we would be able to compute the general direction in which one’s fallacy, if any, went. We called this the “Bias Index (BI),” and it represented the proportion of predictions aligning with either NR bias or PR bias. Predictions consistent with PR were coded as 1, while those consistent with NR were coded as 0. The BI, then, was calculated by dividing the number of PR predictions by the total number of predictions. Therefore, an index closer to 1 indicated a higher tendency to follow PR. On the other hand, an index closer to 0 specified a stronger tendency to show NR. A BI of 0.5 implied that neither cognitive fallacy was dominant. Thus, if our prediction were valid, people would have either a low or high index value in the “same trial condition” but that index value would approach 0.5 in the “different trial condition.” In this task, participants completed eight actual trials randomly in each condition with no time limit (due to the manipulation, in the “different trial” condition, one actual trial was a combination of the two trials).

#### Closure-based coin prediction task (C-CPT)

This task was designed based on the study by Roney and Sansone (2015). All participants completed this task across two closure conditions. In each trial consisting of thirteen coin tosses, they were asked to predict the outcome of each toss. As in Roney and Sansone’s (2015) study, the first eight outcomes (e.g., “HTHHTHHT”) were identical across both closure conditions, but the last four differed. In the “open run” condition, the last four outcomes were presented in a way that kept the run open (e.g., “THHH”). However, in the “closed run” condition, the run was interrupted by a different outcome (e.g., “HHHT”). In this task, we observed whether participants exhibited cognitive fallacies when predicting the thirteenth outcome after observing the twelve-toss results. In both conditions, the twelve outcomes always maintained an 8:4 event ratio (either “eight heads and four tails” or “four heads and eight tails”). Participants completed a total of 18 trials randomly without a time limit: six trials with random stimuli, six for the “closed run” condition, and six for the “open run” condition. For analysis, only the twelve trials for the closure conditions were used.

Given the results from Roney and Sansone’s (2015) study, we predicted that participants would follow the cognitive fallacies in the “open run” condition but not in the “closed run” condition. To examine our prediction, we again computed a BI, following the approach used in G-CPT. If our prediction were valid, participants should exhibit either low or high index values in the “open run” condition, while in the “closed run” condition, their index value should be around 0.5 or close to it.

#### Manipulation checks

To assess perceived relatedness, participants answered two questions (Selimi et al. 2022):

- MC1: “The prior results were related to my prediction.”
- MC2: “I took the previous results into account to make my prediction.”

Responses were made on a 5-point Likert scale (1 = strongly disagree, 5 = strongly agree).

### Participants

A hundred and thirty-three undergraduate students (101 females, 31 males, and one non-binary; M_age_ = 19.98, SD_age_ = 2.463, Range_age_ = 18–34), attending an all-women’s college or the affiliated coed college, were recruited to participate and received course credit for their participation.^2^ All participants signed consent forms prior to beginning the experiment in accordance with IRB-approved guidelines.

We initially believed that at least 30 participants for each bias group (a total of 60) would be required to ensure normality of the data based on the central limit theorem. However, a coin toss is generally known as related to the NR bias, and 70% of participants exhibited NR in the open run condition of Roney and Sansone’s (2015) experiment. Therefore, if we were to recruit only 60 participants, we expected that the distribution of participants’ BI used for bias group classification would be skewed below 0.5, resulting in a relatively small number of participants in the PR group. Considering the distribution of BI associated with the characteristics of the task, we anticipated that recruiting at least 120 participants would ensure that each bias group would indicate at least 30 participants. Consequently, we aimed to recruit approximately 132 participants in total, assuming a dropout rate of 10%.

### Procedure

All procedures were conducted in the laboratory using E-Prime 3.0 software. Participants first responded to questions about demographic information, “Gambling,” and “Probability.” Afterward, they completed a Coin Prediction Task that did not include any manipulations. Once they completed the irrelevant Coin Prediction Task, they were asked to complete the G-CPT and C-CPT. The order of the two tasks was randomized. In each trial of both tasks, participants were asked to predict the outcome of the *N*th coin toss. After their prediction, they saw a short video clip of tossing a half-dollar coin. Then, they received immediate feedback indicating whether their prediction was correct or incorrect. After that, the outcomes of coin tosses up to the *N*th toss were provided, and participants were asked to predict the next toss outcome. The coin toss outcomes were presented as images of a half-dollar coin. In each of the two main tasks (i.e., G-CPT and C-CPT), manipulation checks were conducted upon completion. On average, it took about 13 min to complete each task along with its corresponding manipulation check. After finishing the main tasks and manipulation checks, participants completed the irrelevant Coin Prediction Task again, and then they were debriefed. The overall procedure for this experiment took about 53 min. Throughout all Coin Prediction Tasks, information about the base rate or the fairness of the coin toss was not provided.

## Results

In this study, we set two primary goals of analysis. First, we aimed to examine whether individuals’ tendencies to follow specific cognitive fallacies decreased when events are perceived as unrelated compared to when they are perceived as related. To do so, we created a variable called “Bias group,” which categorized people as those who showed either NR bias or PR bias in conditions where events were perceived as related. Using this variable, we could test our hypotheses by comparing the Bias Index across the two experimental conditions for each task. Second, we aimed to assess the effectiveness of the experimental manipulation in each task. If a specific condition (e.g., “different trial” condition or “closed run” condition) successfully induced the perception that events were unrelated, then manipulation check scores should consistently be lower. To achieve these two goals, we used SPSS 29 for all data analyses.

For analysis, participants were categorized into specific “Bias groups” on their Bias Index in the conditions where past and future events were perceived as related (G-CPT: “same trial”; C-CPT: “open run”). Participants with an index below 0.5 were assigned to the “NR group,” while those with an index above 0.5 were included to the “PR group.” Additionally, participants with an index of exactly 0.5 were placed in the “No fallacy group.” A cross-table between task type and Bias group is presented in Table 1.

One notable finding from Table 1 is the intra-individual variability in cognitive fallacies across tasks. Only 47 participants consistently showed the same cognitive fallacy across both tasks (NR: 15 participants; PR: 32 participants), whereas many of the participants revealed different fallacies across tasks. This variability could be attributed to task-specific sequence characteristics (e.g., sequence length, run length, etc.), though this remains uncertain. Crucially, this intra-individual variability suggests that the tasks should be analyzed separately. That is, the Bias group categorization for each task should be used only for the analysis of each task.

Another interesting observation was that a considerable number of participants were classified into the No fallacy group in at least one of the tasks. In these cases, their Bias Index showed zero variance in specific conditions (G-CPT: same trial condition; C-CPT: open run condition), which violated the assumptions of most statistical tests (e.g., ANOVA). In each task, therefore, individuals in the No fallacy group were excluded from two primary analyses: a test of the effectiveness of manipulation and a test of our hypotheses. However, we conducted additional analyses for these participants to examine whether their Bias Index in the other condition (G-CPT: different trial condition; C-CPT: closed run condition) significantly differed from 0.5.

Across all 133 participants, the mean “Gambling” score was 1.59 (SD = 0.760), indicating that they rarely engage in gambling. Their mean “Probability” score was 2.45 (SD = 1.171), which indicated that they were slightly less familiar with the law of probability.

**Table 1:** Contingency table of bias group by task type

		C-CPT			Total
		NR group	No fallacy group	PR group	
G-CPT	NR group	15	11	6	32
	No fallacy group	6	20	10	36
	PR group	18	15	32	65
Total		39	46	48	133

### Grouping-based coin prediction task (G-CPT)

Summary: The grouping manipulation effectively influenced the perceived relatedness in the manipulation checks. Accordingly, the Bias Index analyses showed that NR and PR were less likely to appear when the previous outcomes and their prediction were included in the different trials, strongly supporting H1 and H2.

In the G-CPT analysis, 36 participants classified into the No fallacy group were excluded from the primary analyses, leaving 97 participants to be included. We first used a paired-sample t-test to test whether the manipulation check scores differed in the grouping conditions (“same trial” vs. “different trial”). The descriptive statistics and test results are shown in Table 2. The difference in MC 1 across the two conditions was significant ($\:t\left(96\right)=\:3.287,\:p<.01$). That is, participants believed that the previous outcome and next outcome were more related in the “same trial” condition than in the “different trial” condition. The difference in MC 2 across the grouping conditions was also significant ($\:t\left(96\right)=\:2.844,\:p<.01$). Participants reported that they used the previous outcomes to predict the next outcome significantly more in the “same trial” condition than in the “different trial” condition. These results indicated that the grouping manipulation was effective.

**Table 2:** Descriptive statistics and significance test results on manipulation checks (G-CPT)

Type	Mean	SD	Significance
MC 1 (Same Trial)	2.98	1.275	$\:p<.001$
MC 1 (Different Trial)	2.64	1.129	
MC 2 (Same Trial)	3.14	1.346	$\:p<.01$
MC 2 (Different Trial)	2.86	1.258	

**Fig. 1:**
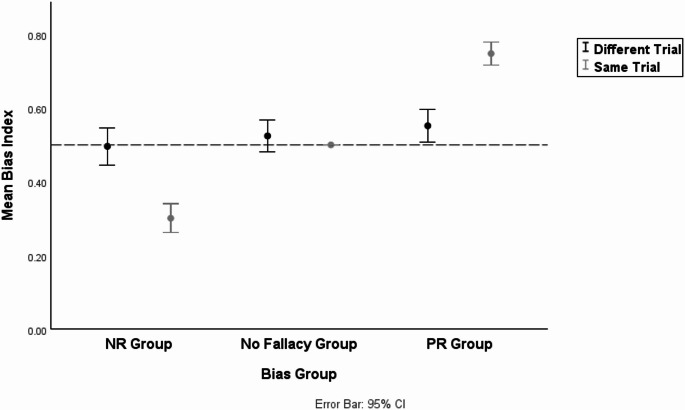
Mean Bias Index by Bias Group and Grouping Condition (G-CPT)

To examine the differences in the Bias Index by the Bias group (NR vs. PR) and the grouping condition (same trial vs. different trial), we conducted a two-way repeated measures ANCOVA. The Bias group was a between-subject factor, and the grouping condition was a within-subject factor. “Gambling” and “Probability” were set as covariates. The descriptive statistics of the Bias Index by the Bias group and the grouping condition are shown in Table 3. The results showed a significant main effect of the Bias group ($\:F=106.671,\:p<.001,\:{\eta\:}^{2}=.53$), which meant that participants in the PR group showed a higher Bias Index than those in the NR group. Importantly, the interaction effect between the Bias group and the grouping condition was significant ($\:F=79.655,\:p<.001,\:{\eta\:}^{2}=.46$). The interaction effect can be seen in Fig. 1. However, the main effect of the grouping condition was not significant ($\:F=0.010,\:p>.05,\:{\eta\:}^{2}<.001$). In addition, the effects of covariates were not significant ($\:Gambling:\:p=.24;Probability:p=\:.86$).

We further conducted pairwise comparisons to test for the simple main effect. In the NR group, the difference in the Bias Index across the grouping condition was significant ($\:p<.001,\:{\eta\:}^{2}=.24$), which meant that participants in the NR group were less likely to follow NR in the “different trial” condition compared to in the “same trial” condition, supporting H1. For the PR group, the difference in the Bias Index across the grouping condition was also significant ($\:p<.001,\:{\eta\:}^{2}=.40$). This indicated that participants in the PR group were less likely to follow PR in the “different trial” condition rather than the “same trial” condition, supporting H2. For the grouping condition, the difference in the Bias Index across the Bias group was significant in the “same trial” condition ($\:p<.001,\:{\eta\:}^{2}=.75$), but not in the “different trial” condition ($\:p>.05,\:{\eta\:}^{2}=.02$).

**Fig. 2:**
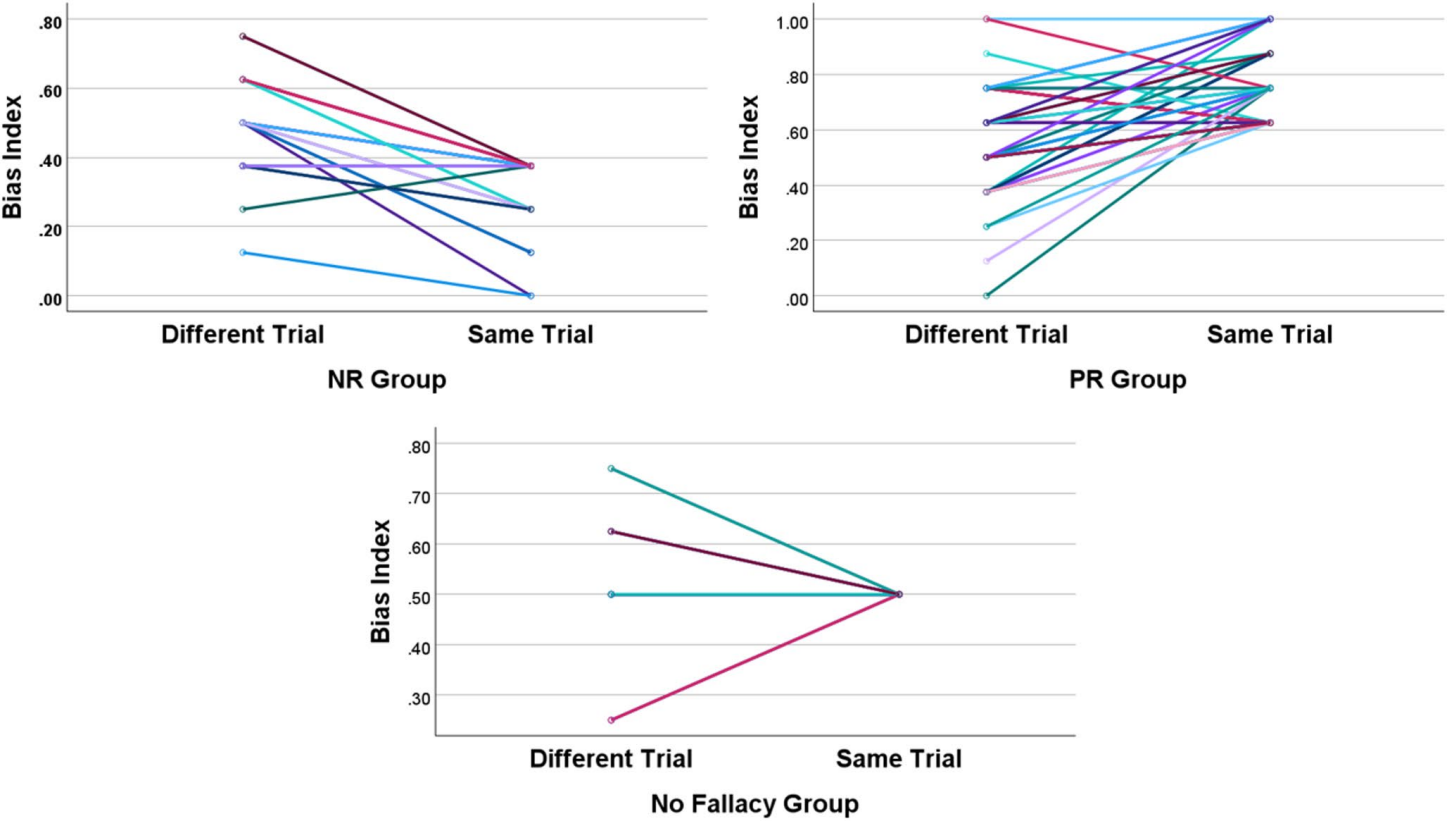
Spaghetti Plots of Individual Bias Indices Across Grouping Conditions (G-CPT)

We additionally conducted a one-sample t-test to investigate whether the BI of the No fallacy group, which was excluded from the primary analyses, differed from 0.5 in the different trial conditions. Descriptive statistics for the No fallacy group are presented in Table 3, and it is visualized in Fig. 1. The results indicated that the BI of the No fallacy group in the different trial condition did not significantly differ from 0.5 ($\:t\left(35\right)=1.156,\:p=.26$). In other words, the No fallacy group did not exhibit any significant fallacy in the different trial condition as well. To visually identify how individual participants’ BI in each Bias group varied across the grouping conditions, we presented spaghetti plots in Fig. 2.

**Table 3:** Descriptive statistics of bias index by bias group and grouping condition

Grouping Condition		*N*	Mean	Median	Standard Deviation
Same Trial	NR group	32	0.301	0.375	0.109
	No fallacy group	36	0.5	0.5	0
	PR group	65	0.748	0.750	0.128
Different Trial	NR group	32	0.496	0.5	0.140
	No fallacy group	36	0.524	0.5	0.126
	PR group	65	0.552	0.5	0.182

Participants in the No fallacy group were excluded from the primary analyses testing the effectiveness of manipulation and the differences in Bias Index across the grouping conditions. Instead, an additional analysis was conducted to examine whether their BI in the different trial condition significantly differed from 0.5

### Closure-based coin prediction task (C-CPT)

We can summarize the findings as follows: The closure manipulation did not affect perceived relatedness in the manipulation checks. However, the Bias Index analyses still showed reductions in NR and PR when runs were closed, partially supporting H1 and H2.

In the C-CPT analysis, 46 participants classified into the No fallacy group were excluded from the primary analyses, and 87 participants were included. We first conducted a paired-sample t-test to verify the effectiveness of the closure manipulation. As shown in Table 4, the differences in MCs by the closure condition were not significant ($\:both:p>.05$), which suggests that the closure manipulation was not effective in the C-CPT. However, this may be because our manipulation checks assessed the perceived relatedness of all prior outcomes and the prediction rather than focusing specifically on the final part of the sequence where the closure manipulation was applied. As a result, participants’ responses might have been influenced more by the grouping than by the closure itself. Therefore, we continued to analyze the data to check whether the differences in the Bias Index across the Bias group and the closure condition were significant.

**Table 4:** Descriptive statistics and significance test results on manipulation checks (C-CPT)

Type	Mean	SD	Significance
MC 1 (Open Run)	3.174	1.121	$\:p=.67$
MC 1 (Closed Run)	3.190	1.127	
MC 2 (Open Run)	3.224	1.138	$\:p=.44$
MC 2 (Closed Run)	3.199	1.151	

**Fig. 3:**
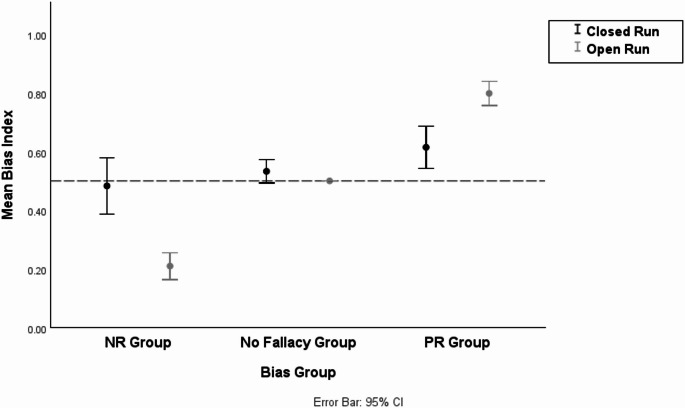
Mean Bias Index by Bias Group and Grouping Condition (C-CPT)

We conducted a two-way repeated measures ANCOVA as we had done with G-CPT. The descriptive statistics of the Bias Index by the Bias group and the closure condition are shown in Table 5. The results showed that there was a significant main effect of the Bias group (open run vs. closed run; $\:F=87.401,\:p<.001,\:{\eta\:}^{2}=.51$), which indicates that participants in the PR group showed a higher Bias Index compared to those in the NR group. In addition, there was a significant interaction effect between the Bias group and the closure condition ($\:F=60.142,\:p<.001,\:{\eta\:}^{2}=.42$). The interaction effect can be seen in Fig. 3. However, the main effect of the closure condition was not significant ($\:F=0.081,\:p=.77,\:{\eta\:}^{2}<.01$).

We additionally conducted pairwise comparisons to test simple main effects. For the NR group, the difference in the Bias Index across the closure condition was significant ($\:p<.001,\:{\eta\:}^{2}=.33$). The participants in the NR group were less likely to follow NR in the “closed run” condition rather than in the “open run” condition, which supported H1. For the PR group, the difference in the Bias Index across the closure condition was also significant ($\:p<.001,\:{\eta\:}^{2}=.21$). This implied that people in the PR group were less likely to follow PR in the “closed run” condition rather than in the “open run” condition, supporting H2. In addition, there was a significant difference in the Bias Index by the Bias group for the “open run” condition ($\:p<.001,\:{\eta\:}^{2}=.81$) and the “closed run” condition ($\:p<.05,\:{\eta\:}^{2}=.06$).

**Fig. 4:**
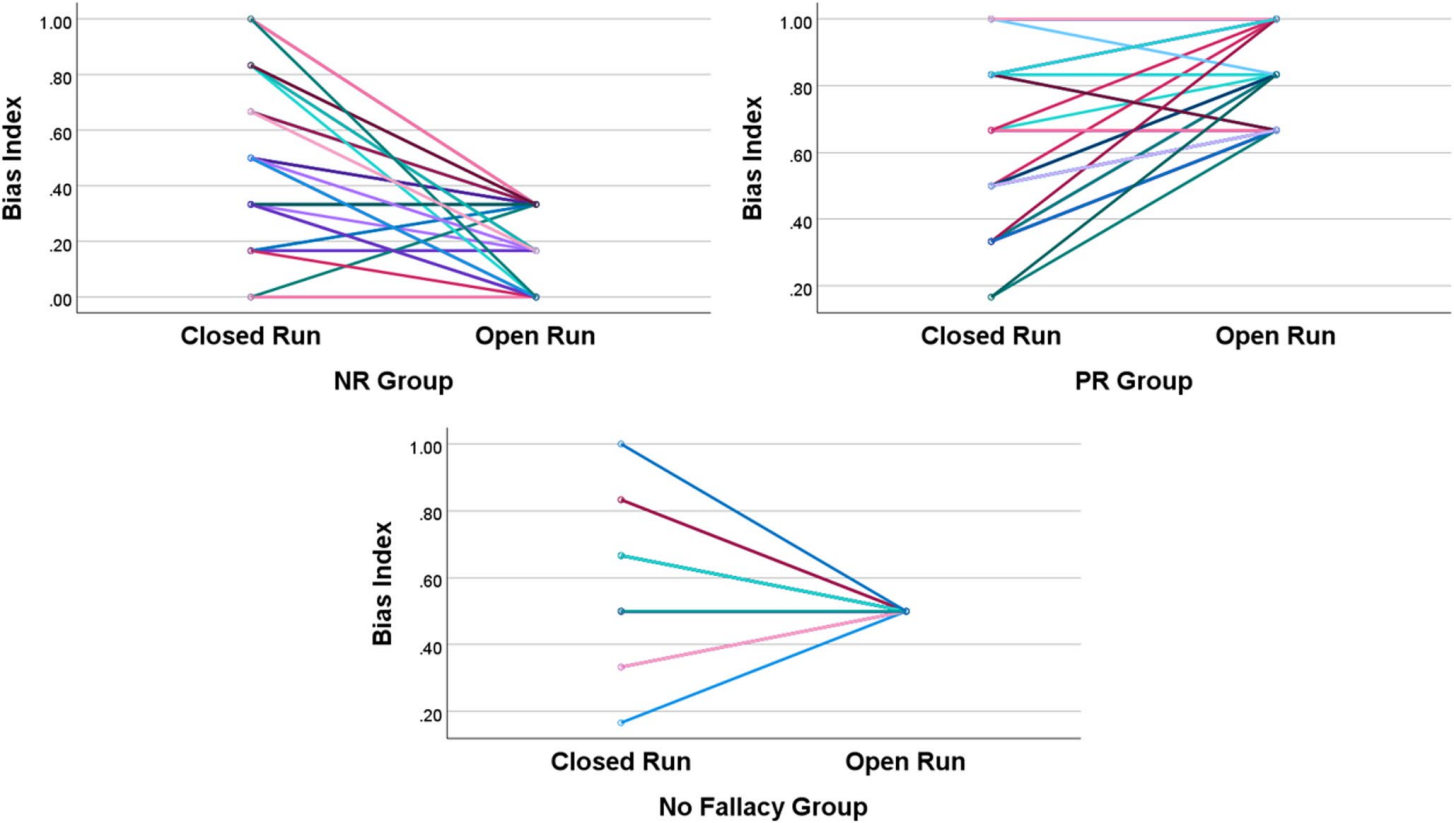
Spaghetti Plots of Individual Bias Indices Across Grouping Conditions (C-CPT)

Furthermore, we examined whether the BI of the No fallacy group differed from 0.5 in the closed run condition. Descriptive statistics for the No fallacy group are presented in Table 5, and it is visualized in Fig. 3. A one-sample t-test showed that the BI of the No fallacy group in the closed run condition did not significantly differ from 0.5 ($\:t\left(45\right)=1.646,\:p=.11$). That is, the No fallacy group did not exhibit any specific BI in the closed run condition either. To visually identify how individual BI in each Bias group varied across the closure conditions, we presented spaghetti plots in Fig. 4.

**Table 5:** Descriptive statistics of bias index by bias group and closure condition

Closure Condition		*N*	Mean	Median	Standard Deviation
Open Run	NR group	39	0.209	0.333	0.142
	No fallacy group	46	0.5	0.5	0
	PR group	48	0.799	0.833	0.142
Closed Run	NR group	39	0.483	0.5	0.296
	No fallacy group	46	0.533	0.5	0.134
	PR group	48	0.615	0.5	0.248

Participants in the No fallacy group were excluded from the primary analyses testing the effectiveness of manipulation and the differences in Bias Index across the closure conditions. Instead, an additional analysis was conducted to examine whether their BI in the closed run condition significantly differed from 0.5

## General discussion

The present study explores how perceptual organization processes influence two cognitive fallacies. Unlike prior research, we verified through manipulation checks whether Gestalt-based manipulation altered an individual’s perception of the relatedness of events. Our findings extend previous evidence that negative recency (NR) can be reduced by Gestalt principles to the domain of positive recency (PR). This study suggests that perceptual organization should be considered to understand the fallacies in probabilistic decision-making contexts. The following sections present the specific objectives, results, and discussion of the current study.

In this study, we investigated (1) whether Gestalt-based manipulations could lead individuals to perceive past outcomes and a future event as unrelated and (2) whether these manipulations could indeed reduce their tendencies to follow NR or PR. In the Grouping-based Coin Prediction Task (G-CPT), we found that when past outcomes and a prediction for the 7th coin toss were presented within the same trial, participants recognized the past results as more relevant to their prediction for the 7th toss. As a result, they were more likely to exhibit NR or PR. In contrast, when the prior outcomes and the prediction for the 7th toss were presented in separate trials, they viewed these events as less interrelated, and accordingly, their tendency to follow either NR or PR was reduced. These findings supported our hypotheses (H1 & H2). Similarly, in the Closure-based Coin Prediction Task (C-CPT), we discovered that the closure manipulation – altering the final part of the previous outcomes – affected individuals’ tendency to follow NR or PR. When the recent preceding outcomes were a streak of the dominant event (e.g., “THHH”), participants tended to follow NR or PR. On the other hand, this tendency weakened when the final previous outcome reflected a non-dominant event (e.g., “HHHT”). While these findings align with our predictions, participants ironically perceived that the prior outcomes and their predictions were related in both conditions. In other words, although the closure manipulation could reduce the tendency to exhibit cognitive fallacies, it failed to induce a perception of unrelatedness.

The findings from the C-CPT suggest two possible interpretations. First, it is possible that the closure manipulation worked as intended, but our manipulation checks failed to detect this effect. In the manipulation checks, because we assessed perceived relatedness between “all” prior outcomes and the subsequent prediction, participants may have responded based on the grouping between them rather than the closure manipulation. To assess the effect of the manipulation more directly, it would be recommended to focus only on the final part of the previous outcomes where the manipulation was applied. For example, instead of asking participants to evaluate their agreement about whether “the prior coin toss results (1st – 12th ) were related to my 13th toss prediction,” a more targeted statement such as “the last four of the prior coin toss results (9th – 12th ) were related to my 13th toss prediction” might better capture participants’ perception of unrelatedness under closure manipulation. Secondly, it could happen that the closure manipulation did not influence participants’ perception of relatedness and that the observed reduction in the tendency of cognitive fallacies may instead be attributed to whether they responded to the final part of the previous outcomes. For instance, when the prior results ended with a non-dominant outcome (e.g., “HHHT”), most participants might predict tails (NR) or heads (PR) based on the streak (“HHH”). However, some of them might anchor their prediction on the final outcome “T,” and they would forecast heads (NR) or tails (PR). Since our BI was coded based on the streak, the final outcome-based predictions might be coded as inconsistent with the expected fallacies. As a result, this could raise the false result that participants were less likely to follow NR or PR when previous outcomes ended with the non-dominant event. It remains unclear which of the two possibilities is more likely, but further research could clarify this issue by implementing more refined manipulation checks.

On the other hand, our findings from the C-CPT can be considered in an evolutionary context. For example, in a foraging context, it is a crucial survival strategy for animals to remain in resource-rich patches (Blanchard et al. 2014; Wilke and Barrett 2009). In such cases, the frequency of rewarding outcome (e.g., successful hunting) may be a more important cue than the continuity of those outcomes, and leaving the area after a single non-rewarding outcome (e.g., failed hunting) could be maladaptive. In a similar way, people may make predictions in the closed run condition based on the frequency of a particular outcome. However, this logic does not seem to apply to probability games such as coin toss and baccarat. In the C-CPT, the frequency of heads and tails was always controlled to be equal across the two closure conditions (e.g., eight heads and four tails). If frequency had been a useful cue in the task, then the differences in BI between the two closure conditions should not be significant. In baccarat, similarly, Muto et al. (2025) reported that when a specific outcome appeared in a row and was considered “hot,” people stopped betting on it once the streak was broken. These findings suggest that, unlike in foraging contexts, frequency information does not have a strong influence in closed runs.

One natural question that arises from the current study is why participants exhibited PR in the Coin Prediction Tasks. A coin toss is typically considered a representative example of random events, and people are generally known to follow NR in random events (Ayton and Fischer 2004; Burns and Corpus 2004; Oskarsson et al. 2009). However, Rao and Hastie (2023) demonstrated that NR is consistently observed in randomly generated events only when the base rate is known. In contrast, when the base rate was unknown or uncertain, individuals tended to show PR-like judgments as streak length increased – even in events generated by random processes. In other words, people may show PR in random events when reliable base-rate information is not provided. In all tasks used in the current study, we did not provide participants with explicit base-rate information about the coin tosses. Thus, the co-occurrence of both NR and PR in our tasks may reflect a combination of participants’ stereotypical beliefs about coin tosses and their sensitivity to local streaks within the presented sequences. As this could not be checked in the current study, we hope that future research might establish experimental conditions that reliably elicit NR and PR in order to test the effectiveness of Gestalt-based manipulations.

Finally, we note the limitations of our research, suggesting directions for future research. First, 75.9% of our sample consisted of female participants. We acknowledge that this imbalance limits the generalizability of our findings. Future research should therefore aim to achieve a more balanced sample in terms of gender. Second, our study was limited to the coin toss paradigm. Coin tosses are typically associated with NR rather than PR (Dohmen et al. 2009). As a result, our investigation of NR and PR was restricted to the level of individual differences in the coin toss paradigm. To investigate whether the Gestalt-based manipulation is effective for both fallacies, it would be necessary to test it in contexts where PR is more prevalent (e.g., sports). Third, due to the random variability of the BI, some participants may be misclassified into the wrong bias groups despite the BI being designed to reduce such errors. For instance, an individual who generally exhibits PR could, by chance (e.g., through guessing), make a prediction reflecting NR and thus be misclassified into the NR group. To address this problem, future research could use more sequences or ask participants which cues or strategies (e.g., frequency, streak, and guessing) guided their predictions. In the latter case, predictions associated with cues such as a “streak of an outcome” could be incorporated into the BI, whereas predictions attributed to “guessing” could be excluded from the BI.

## Conclusion

Overall, the findings demonstrate that Gestalt principles of grouping and closure shape the extent to which people succumb to cognitive fallacies. By showing that both NR and PR are reduced when outcomes are perceptually separated or closed, this study provides new evidence for the perceptual underpinnings of reasoning errors. More broadly, these results emphasize the value of considering perceptual organization processes in models of judgment and decision-making.

## Data Availability

Data is available at https://osf.io/ynbsu/?view_only=a5b511e21fe9486796dcaf61044b5b96. Additional materials (e.g., E-Prime experiment files) are available upon request.

## Footnotes

1 Positive recency has often been equated with the hot hand fallacy. However, the hot hand fallacy can be applied in contexts where an individual’s streak of success is presented, whereas positive recency can be applied in any context where either an individual’s streak of success or the streak of an outcome of an event is presented (Croson and Sundali 2005; Muto et al. 2025; Sundali and Croson 2006).

2 A response of 0 for age was treated as missing, and one participant was therefore excluded from the descriptive statistics of age.
